# News and Events of the Week

**Published:** 1909-11-20

**Authors:** 


### News and Events of the Week.

Dr. Philip Bahr and some students of the London School for the Study of Tropical Medicine left for Fiji to study dysentery, on November 19. The money for the expedition is being provided by the family of Lord Sheffield.

The Surrey County Council has decided to terminate the agreements with the district medical officers of the county as inspectors of midwives under the recent Act, and has appointed Miss Falk, the superintendent of the Surrey Nursing Association, to perform the duties at a salary of £120 a year.

Switzerland is suffering as much as ever from the effects of goitre, "the pest of the Alps." The military statistics show that the Swiss Republic loses the service of between 2,200 and 2,500 young men every year from this disease. The two most mountainous centres, Valais and Grisons, are most subject to its prevalence. In Grisons, in fact, 4 per cent. of the school children have been attacked by it. It is hoped that preventive measures may be adopted by the Federal Government.

The directors of the Eye Infirmary, Glasgow, have appointed Dr. Arthur J. Ballantyne, surgeon to the infirmary, to fill the vacancy caused by the death of Dr. T. S. Meighan. Dr. Ballantyne, who is a native of Glasgow, graduated in medicine in 1898, and took the degree of M.D. in 1901. He is a Fellow of the Faculty of Physicians and Surgeons of Glasgow, and holds the appointment of Professor of Physiology in Anderson's College. He studied for some time in Vienna. Dr. Ballantyne has made various contributions to the ophthalmological journals.

The General Council of Glasgow University has approved the proposed new arrangements for clinical teaching. The scheme is intended to utilise to the full the general hospitals of Glasgow for clinical teaching. By a majority the Business Committee approved generally of its terms, regarding the scheme as calculated to benefit the students who resorted to the University for medical education and to redound to the credit of the Medical School of Glasgow. The value of the scheme was felt to be enhanced by the fact that it was capable of being extended to include the Victoria Infirmary as well as the Royal Infirmary. An offer has been made to provide the necessary funds for the endowment of four teachers of professorial rank to the amount of £1,800.

At a public meeting held in the National Schools, at Didsbury, Manchester, it was decided to raise funds for a memorial to the late Dr. J. Milson Rhodes. Dr. Rhodes was an active member of the Executive Council of the Poor Law Unions Association for nearly thirty years, and many of his friends in various parts of the country will, no doubt, like to contribute to the memorial. He was a member of the Provisional Committee appointed to bring the Association into being in 1898; he was Vice-President in 1899, and President in 1900 and 1901. He was from the beginning intimately connected with the Parliamentary work of the Association, and he was a member of the Parliamentary Committee from 1899 to 1901, and from 1905 until his decease. The Chairman of the Committee is Mr. Fletcher Moss, J.P.; Mr. Jas. H. Norris, 7 Wilmslow Road, Didsbury, is Hon. Treasurer, and Messrs. Thos. Spann, 37 Wilmslow Road, Didsbury, and J. T. Seale, 8 Spring Gardens, Didsbury, Hon. Secretaries.

At the quarterly meeting of the trustees of the Hunterian Collection, held on November 10 at the Royal College of Surgeons, it was decided that the election of a trustee in the vacancy occasioned by the death of Sir Thomas Smith should take place at the meeting of the Board next February.

At the last meeting of the Council of the Royal College of Surgeons the death of Mr. Henry Hugh Clutton, a member of the Council, was reported. A resolution was adopted expressing regret at the loss and sympathy with the members of his family, and recording its appreciation of his services to the college during the past seven years. The vacancy on the Council thus occasioned will be filled up at the annual meeting of Fellows in July 1910.

At the last meeting of the Hanley Education Committee a letter was read from the Secretary of the local branch of the British Medical Association informing the Committee that the salary attached to the post of local school medical officer was inadequate, since it is below the minimum of that approved by the Association for appointments of this nature—£250—and threatening to issue a warning notice to all candidates against applying for the post unless the salary was raised. After some discussion it was decided to advertise for a lady medical officer at £200 a year.

The following letter, referring to the late Dr. J. H. Wells, appeared in the *Times* of November 12:—

The death of Dr. John Herbert Wells last month at the early age of thirty adds another name to the list of those who have lost their lives in the cause of scientific medical research.

After distinguishing himself in original research, Dr. Wells joined the department of therapeutic inoculation at St. Mary's Hospital, where this branch of treatment was advanced materially by his labours. In February 1908 Dr. Wells undertook pioneer investigation of the treatment of glanders, and in the course of laboratory work, which resulted in saving the life of the patient from this, till then, hopeless disease, contracted infection himself and died after eighteen months of suffering, on October 16, 1909, leaving a widow and two small children almost totally unprovided for. . . .

A committee has been formed, consisting of the Right. Hon. A. J. Balfour, the Earl of Dalhousie, Lord Justice Fletcher Moulton, Sir Almroth Wright, M.D., F.R.S., and Messrs. H. A. Harben (late Chairman of St. Mary's Hospital), Julian G. Lousada, Edmund Arthur Smith (of the Stock Exchange), and Dr. W. H. Willcox, and they now appeal to the public for donations to a fund to be held in trust for Dr. Wells's widow and children. The Committee (on whose behalf we write) feel strongly that where a man has laid down his life in trying to relieve the sufferings of mankind, it is eminently fitting that some recognition should be made of his work, and they have full confidence that the public will make a ready response to their appeal.

All contributions sent to the honorary secretaries and treasurers of the fund, at 16 Old Broad Street, London, E.C., will be gratefully acknowledged.

Yours faithfully,

Dalhousie, Julian G. Lousada — Joint Hon. Secretaries and Treasurers.

16 Old Broad Street, London, E.C., November 11.

A meeting of the medical men of Chelsea and Fulham was held at the Fulham Town Hall on Wednesday, November 10. The object of the meeting, which was regarded as the first organised attempt of the medical profession to support its claims against the public authorities of London, was to protest against the refusal of the Board of Guardians to guarantee certain medical fees due from the poor of the district for services rendered. The Chairman said that the meeting was a sequel to the refusal of responsibility by the Guardians for payment of medical fees in a number of maternity cases when emergency medical calls were made by midwives. Dr. Edwards, a member of the Board of Guardians, stated, however, that since the Local Government Board Order of 1907 they had had but forty-nine cases locally, and in only seven of these had payment been refused.

